# 慢病毒载体及其研究进展

**DOI:** 10.3779/j.issn.1009-3419.2014.12.09

**Published:** 2014-12-20

**Authors:** 琛 陈, 海粟 万

**Affiliations:** 300052 天津，天津医科大学总医院，天津市肺癌研究所，天津市肺癌转移与肿瘤微环境实验室 Tianjin Key Laboratory of Lung Cancer Metastasis and Tumor Microenviroment, Tianjin Lung Cancer Institute, Tianjin Medical University General Hospital, Tianjin 300052, China

**Keywords:** 慢病毒载体, 病毒包装, RNA干扰, 应用, Lentiviral vector, Virus packageing, RNA interference, Application

## Abstract

慢病毒载体（lentivirus vector, LV）是目前分子及细胞生物实验中非常有效的工具，在基因转染方面有着许多独特的优势，例如，对细胞是否处于有丝分裂期没有特别的要求，基因转染效率高，可容纳较大基因片段等。本文将就慢病毒载体的来源、分子特征及研究进展等进行综述。

## Abstract (en)

Lentiviral vectors are currently very effective tools in molecular and cell experiment. Lentiviral vector, a kind of retroviral vectors, has a number of unique advantages in target gene transferation, for example, the ability of transfection to the dividing or nondividing cells, its high efficiency of transfection and a capacity of large target gene fragments. This paper describes the sources of lentiviral vectors, molecular characteristics, research progress, *etc*.

慢病毒载体作为一类来源于逆转录病毒的载体，以其转染效率高，可感染分裂期和非分裂期细胞，可容纳较大的基因片段等优点，有着广泛的应用前景^[1-4]^。

## 1 慢病毒载体的来源

慢病毒属（lentivirus）在分类上属于逆转录病毒科，为二倍体RNA病毒。原发感染的细胞以淋巴和巨噬细胞为主，导致感染个体发病。慢病毒感染的主要临床特点是在出现典型的临床症状以前，经历较长的潜伏期，之后缓慢发病，因此被称为慢病毒。而慢病毒载体则是以慢病毒的基因组为基础，将其中多个和病毒活性相关的序列结构去除，使其具有生物学的安全性，然后，再在这个基因组骨架中引入实验所需要的目标基因的序列和表达结构，并将之制备成载体^[5]^。早期的慢病毒载体，是由人类免疫缺陷病毒（human immunodeficiency virus, HIV）改装而来，包括HIV-1型载体系统和HIV-2型载体系统。

后来随着研究的深入，研究人员也开发了许多其他类型的慢病毒载体系统，主要包括猿类免疫缺陷病毒（simian immunodeficiency virus, SIV）载体系统、猫免疫缺陷病毒（felines immunodeficiency virus, FIV）载体系统、马传染性贫血病毒（equine infectious anemia virus, EIAV）载体体系、山羊类关节炎-脑炎病毒（caprine arthritis-encephalitis virus, CAEV）载体系统、牛免疫缺陷病毒（bovine immunodeficiency birus, BIV）载体系统、绵羊髓鞘脱落病毒（visna/maedi virus, VMV）载体系统等。显然，来源不同物种的载体系统可能会更有效的在相应物种细胞进行基因转染实验，而是否可以在不同物种类型细胞中交叉使用慢病毒载体，特别是是否可以将其他物种的慢病毒体系用于人类，还需要进一步研究和评估^[6, 7]^。HIV载体系统使用非常广泛，以下的内容将主要以HIV-1为例子对慢病毒载体系统进行介绍。

## 2 慢病毒载体系统的建立

如前所述，慢病毒属于反转录病毒。HIV-1为双链RNA病毒，它的基因组结构（图 1）和其他的反转病毒类似，含有可编码三种主要病毒结构蛋白的基因，即*gag*、*pol*和*env*，*gag*基因编码病毒的核心蛋白如核衣壳蛋白（p7）、内膜蛋白（p17）和衣壳蛋白（p24）；*pol*基因编码病毒复制相关的酶；*env*基因编码病毒包膜糖蛋白。此外，病毒基因组还编码两个调节蛋白，即*tat*、*rev*，*rev*主要参与蛋白调节的表达水平，*tat*参与蛋白转录的控制，与病毒的长末端重复序列（long terminal repeats, LTRS）结合后促进病毒的所有基因的转录。以及四个辅助蛋白，即*vif*、*vpr*、*vpu*、*nef*^[8, 9]^。在HIV-1的基因组结构上，还含有病毒生命史所需要的其他序列结构，例如病毒复制和包装等所需要的信号等。

**1:**
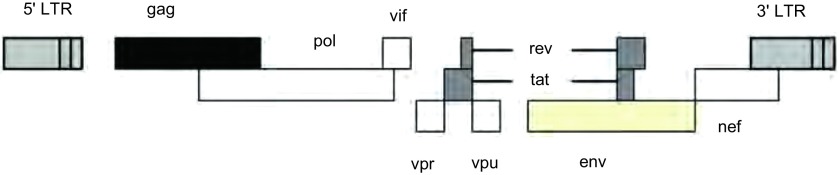
*HIV-1*的基因结构 Structure of human immunodeficiency virus (HIV)-1

HIV-1型慢病毒载体系统的建立，经历了一个逐步完善的过程。其主要的目的，是提高载体的生物安全性。这主要分为以下四个阶段:

第一代是两质粒系统，以HIV-1为骨架构建起来的反转录病毒载体，包含3个结构基因*env*、*gag*和*pol*，2个调控基因*tat*、*rev*和4个辅助基因*vpr*、*vif*、*vpu*和*nef*，两端有长末端重复序列（long terminal repeat, LTR），5' LTR和*gag*之间还有病毒包装信号序列。慢病毒载体最初的包装是将HIV基因组中的反式作用蛋白基因序列去除，然后包装上含有目标基因的重组载体和能够反式提供病毒颗粒所需蛋白的包装质粒，同时共转染包装细胞（如293T细胞）进行包装。这是一个复制缺陷型的载体体系，产生病毒的滴度很低，而且只能感染CD4^+^细胞，在转染过程中包装的蛋白DNA和载体DNA有重组的可能，进而有产生有复制能力病毒（replication competentvirus, RCV）^[10, 11]^，具有产生活性HIV病毒的很大风险。

第二代的三质粒表达系统，也就是将HIV-1基因组中负责包装，逆转录和整合所需要的顺式作用序列结构和编码反式作用蛋白的序列分离，再克隆到三个独立的质粒中，其中一个是包装质粒，含有CMV启动子，控制除*env*的所有病毒结构基因的表达，第二个是包膜质粒，含有表达水泡性口炎病毒糖蛋白（*VSV-G*）基因的序列，这个基因用于代替原病毒的*env*基因，这个基因的产物，可以提高病毒的宿主范围，而另一个则是载体质粒，其中含有研究人员所感兴趣的基因序列结构。但这个三质粒系统还是保留了HIV-1的附属基因，这个质粒体系产生意外而导致出现具有活性的病毒的可能较大，因而被认为安全系数较低。

第三代质粒系统，为避免第一代质粒系统的安全风险，研究人员对第二代的三质粒系统也做了进一步改进，去除了HIV病毒所有辅助因序列，HIV原有的9个基因保留3个（*gag*、*pol*、*rev*）于构建的慢病毒载体中。体系产生意外重组的可能性很低。

第四代质粒系统，也是四质粒系统，这也是目前被广泛使用的慢病毒载体系统。这个系统是在第三代三质粒系统的基础上改进而来的。是将*env*基因放在一个单独的表达质粒上，此外，还将*tat*基因去除，这样就形成了四个质粒的系统，即pGag/Pol、pRev、pVSV-G，此外，还有一个可以放置目的基因序列的载体。这个体系意外产生活性病毒的可能被大大降低。这类载体系统中含有可诱导性基因，最具代表性的为四环素-诱导系统，其为可调控系统与慢病毒载体的结合产物，人为地控制植入LV的目的基因的表达，使基因的条件性表达和基因敲除都成为可能，并且继续保留了自身失活的优势^[12-16]^，为探索基因的功能提供了有利的工具。

## 3 慢病毒载体的优势和安全性

慢病毒是一类逆转录病毒的总称，由慢病毒改建而来的慢病毒载体因其高效稳定的转移效率成为常用研究工具。与其他逆转录病毒相比，慢病毒有其独特的优点：①较其他逆转录病毒有着更广泛的宿主，对于分裂和非分裂细胞均具有感染能力，对于一些较难转染的细胞，如原代细胞、干细胞、不分化的细胞等，能大大提高目的基因转导效率，使目的基因整合到宿主细胞基因组的几率大大增加。这一特性使得慢病毒载体可以用于采用基因治疗方式治愈疾病的临床试验和研究中。比如一些神经系统方面的疾病。在神经系统中，大部分的神经元和胶质细胞处于一个相对静止的状态，使得其他的逆转录病毒载体对于神经系统方面的疾病的基因治疗并不适用，而实验证实，慢病毒载体无论对于体外的组织和细胞培养试验，还是体内的基因治疗实验，对于神经元和神经胶质细胞都具有较高并且较稳定的转染能力^[17-21]^。②慢病毒载体携带并整合进入宿主细胞的目的基因对转录沉默作用有一定的抵抗能力，可以在靶细胞中得到持续高效稳定的表达。③慢病毒载体中可以插入组织细胞特异性的启动子和增强子，提高转入基因的转录靶向性，使慢病毒载体中的目的基因在特定的组织细胞中表达。④经过构建后的慢病毒载体可以携带大约5 kb，甚至更长的目的基因，因此除了外源的short-hairpin RNAs（shRNAs）等小分子外，很多cDNA也能被克隆进入慢病毒载体，当然随着目的基因长度的增加，其病毒滴度也随之下降^[22-27]^。由于慢病毒载体具有如上众多的优势，使得慢病毒载体成为一种能实现外源基因的高效导入及应用于疾病的基因治疗等多方面的有效工具，具有良好应用前景。

虽然慢病毒载体与其他载体相比具有以上优势，但也有其限制性，主要是生物安全性和病毒滴度两方面。由于慢病毒载体主要来源为HIV病毒，特别是HIV-1应用的较为广泛和深入，因此慢病毒的生物安全性成为使用者担心的主要问题之一。慢病毒的分子结构被不断改进，主要致力于如何阻止形成有复制能力的HIV病毒，现在应用比较广泛的是三质粒、四质粒表达系统，该系统中的包装信号被删除，包装序列不能整合至病毒基因组中，因此病毒感染宿主细胞后不能复制，也不会利用宿主细胞产生新的病毒颗粒。所以我们常用的慢病毒载体中的毒性基因已经被剔除并被外源性目的基因所取代，属于假型病毒^[9, 28-32]^。但是由于HIV-1慢病毒载体的自然宿主是人，则病毒仍然具有可能的潜在的生物学危险，建议不要使用编码已知或可能会致癌的基因的假型病毒，除非已经完全公认某个基因肯定没有致癌性，否则均不建议采用假型病毒进行生物学实验^[33, 34]^。要使慢病毒基因广泛应用于研究和临床，还需要从以下几点着手：一是设计载体时需要进一步缩短载体及包装质粒的病毒编码序列，降低病毒基因重组的几率；二是提高其病毒滴度，使其更加广泛和便利的应用。

## 4 不同公司的慢病毒载体介绍

近几年来，慢病毒载体因其独特的优势被大家所熟知和应用。市场上畅销的慢病毒表达载体主要来自Life Technologies、Clontech，国内还有吉凯基因、辉骏生物、赛业生物、百恩维生物等。

Life Technologies公司的Life ViraPower Lentiviral Expression System是市场上较早的慢病毒表达系统。该系统中共包含4个质粒：一是插入pLenti表达载体，用于插入目的基因，上面包括ψ包装信号以及截短的HIV 3'及5'LTR，便于病毒包装；二是pLP1质粒，表达形成慢病毒结构所必需的*gag*基因以及病毒复制和整合必需的*pol*基因；三是pLP2质粒，用以表达*Rev*蛋白，它能与pLP1上的反应元件共同作用诱导*gag*和*pol*表达，并指导病毒RNA的核运输；四是pLP/VSVG表达*VSV-G*，使宿主范围更广。必须这4个质粒共同作用，才能产生有感染能力的病毒。

现在该公司产品中应用较为广泛的是Life Technologies^TM^在ViraPower^TM^慢病毒蛋白表达系统基础上开发的新型病毒表达系统——ViraPower^TM^ HiPerform^TM^慢病毒蛋白表达系统，其特点为高表达，高低度，快速克隆和易检测。该系统中还增加了两个元件，分别为来源于旱獭肝炎病毒的转录后调控元件（Woodchuck hepatitis virus post-transcriptional regulation element, WPRE）和来源于HIV1整合酶基因的central Polypurine Tract（cPPT），WPRE有利于增加蛋白的表达量，而cPPT元件则有利于增加病毒的滴度，WPRE和cPPT元件能使蛋白的表达量至少提高4倍。同时，为满足不同实验者的实验需求，目前Life Technologies^TM^还可提供组成型的ViraPower^TM^ HiPerform^TM^慢病毒蛋白表达系统，可调控外源基因表达的四环素诱导的ViraPower^TM^ HiPerform^TM^慢病毒蛋白表达系统以及可实现组织或细胞特异性表达的无启动子的ViraPower^TM^ HiPerform^TM^慢病毒蛋白表达系统等。

Clontech公司的Lenti-X^TM^ HTX Packaging System属于第四代包装系统。与Invitrogen第四代的表达载体相似，有ψ包装信号和LTR，也有WPRE和cPPT两个元件。不同之处是Lenti-X^TM^ HTX表达系统将*pol*基因分离出来，使*gag*、*pol*和*env*成为三个独立载体，而不是两个载体，这意味着要产生原始的病毒，还需要更多的重组步骤，进一步提高了生物安全性；并且引入了四环素诱导表达技术，极大地提高了病毒包装所需蛋白的表达量，使获得的病毒滴度更高。则完整的系统包含5个载体，安全性更高。同时试剂盒中的packaging mix也有区别，Lenti-X HT包装系统利用了反式激活级联反应来高水平表达病毒蛋白。HT即high Titers的缩写。一般来说，慢病毒表达的滴度约为10^5^-10^6^ TU/mL，而Clontech的滴度达到10^8^ TU/mL。这就意味着不必浓缩和纯化，就能直接感染细胞。Lenti-X系列还包含了荧光表达载体，使目的蛋白可以在N端或C端融合AcGFP1（绿色）或DsRed（红色）荧光蛋白，这样就很容易观察到蛋白的表达和转运。

吉凯基因也是知名度较高的慢病毒表达系统的公司之一。吉凯基因慢病毒载体系统包括GV慢病毒载体系列，pHelper 1.0载体和pHelper 2.0载体三个质粒。GV慢病毒载体中含有HIV的基本元件5'LTR和3'LTR以及其他辅助元件，例如WPRE（woodchuck hepatitis virus posttranscriptional regulatory element）。通常可以根据不同的实验目的针对GV载体改造，获得带有特定基因序列的慢病毒颗粒，进行不同目的的实验，以满足不同的实验需求。pHelper 1.0载体中含有HIV病毒的编码主要结构蛋白的*gag*基因；编码病毒特异性酶的*pol*基因；编码调节*gag*和*pol*基因表达的调节因子的*rev*基因。pHelper 2.0载体中含有单纯疱疹病毒来源的*VSV-G*基因，提供病毒包装所需要的包膜蛋白。吉凯基因慢病毒产品包括慢病毒包装、慢病毒载体构建、RNAi慢病毒、过表达慢病毒、microRNA慢病毒等。可满足不同的实验需求。

当然慢病毒载体的公司不只以上介绍的几家，现在各个公司的慢病毒表达系统都比较成熟，各有其优点及局限性。没有任何一件产品能够满足所有的实验需求，挑选哪个公司的哪种产品，还需要实验人员充分了解实验产品的性质，优点及其局限性，同时结合自己的实验目的来选择合适的试剂盒。

## 5 慢病毒载体在基因分子生物学研究中的地位和应用

慢病毒载体由于其自身优势，得到了越来越多的认可及应用。比如联合RNA干扰（RNA interference, RNAi）进行实验研究、基因治疗以及细胞和动物模型建立等领域。

### 5.1 慢病毒载体介导的RNAi

在RNAi的应用过程中，慢病毒载体已被作为一个高效实用的基因转移工具在基础和应用研究领域得到广泛应用^[35, 36]^。RNAi是指正常生物体内抑制特定基因表达的一种现象，在进化过程中高度保守的、由双链RNA（double-stranded RNA, dsRNA）诱发的、同源mRNA高效特异性降解的现象。其利用RNA介导，特异性地阻断和降低目的基因的表达。质粒介导RNAi转染率低、基因抑制表达弱、持续时间短、不适合体内实验等缺点，但LV-RNAi作用持久，同时载体感染细胞的范围扩大，既可用于细胞特定基因功能的研究，还可用于基因治疗，并且两种技术的优势结合，已成为肿瘤性疾病治疗一种全新方法，通过LV将肿瘤的治疗基因安全地转移到靶细胞内，在骨肉瘤、白血病、肝癌、鼻咽癌等种恶性肿瘤中的基础研究中均有广泛的应用。Pfeifer等^[37]^利用LV与RNAi技术，将LV介导的siRNA导入小鼠受精卵中，培育后得到特定基因表达沉默的小鼠个体，用于有关疾病机制的基础研究和特定基因缺失后对细胞功能的影响研究等领域。

### 5.2 在肿瘤治疗中的应用

癌症的发生主要与基因突变有关，尤其是与癌基因的激活有关，主要有基因突变、扩增和染色体重排3种激活机制。现在主要通过手术、放疗和化疗等手段进行治疗。基因治疗作为一种新的方法正处于快速发展阶段，主要途径是把目的基因导入靶细胞。针对肿瘤发生发展过程中的异常基因，引入有治疗价值的基因片段，并使其在目的细胞中有效、长久地表达，从而达到治疗目的^[38-42]^。而LV的一些生物学特性使基因治疗的设想具备了可能性。以前列腺癌细胞为研究对象，Yu等^[43]^把一受雄性激素调控并在前列腺特异表达的启动子和另一*EGFP*基因插入LV中，并分别转染正常细胞和前列腺癌细胞，结果只在癌细胞中有目的基因*EGFP*表达，且雄性激素可以促使*EGFP*的表达而不改变表达特异性。从以上实验研究可以看出，如果LV应用于肿瘤疾病的临床治疗，可能会为疾病的治疗带来新的进展。

### 5.3 在神经系统疾病中的应用

慢病毒载体能够稳定转染大多数非分裂的原代细胞，包括神经元，进而能够持续稳定的表达，起到治疗的作用。已有大量研究工作^[44-46]^表明应用慢病毒载体表达多种神经系统疾病的相关基因，在帕金森病、老年痴呆症、脊髓受损等多种疾病的动物模型中进行长期治疗，已取得良好的效果。Poeschla等^[47]^构建慢病毒载体转染于患有帕金森病（Parkinson' s disease, PD）大鼠，结果显示可改善大鼠的旋转次数，保护并防止多巴胺神经元退化，可起到减轻PD神经症状的效果。

### 5.4 转基因动物模型构建

慢病毒载体技术是培育转基因动物模型的有效工具之一，将目的基因转入宿主细胞后，经过反转录、整合等，能高效地使优势基因在宿主体内得到良好的表达。从而得到需要的带有目的基因的个体。由于其操作简单，对宿主细胞有良好的适应性，所以，慢病毒系统在转基因动物方面有着良好的应用前景。许多情况下，这些突变动物表型与某种人类疾病的临床症状类似，使这些突变动物可能成为人类疾病的理想模型，通过转基因和基因打靶获得的遗传工程动物的疾病表型能在近交系中保持高度稳定，与改变营养条件、药物作用或手术途径制备的模型相比，这些遗传修饰动物模型具有更好的一致性和稳定性，能够更加真实地反映人类疾病的病理过程和分子改变^[16, 48-51]^。无数研究^[52-54]^已经证明，许多遗传工程动物（如遗传工程小鼠和小型猪）已成为研究人类重大疾病，包括新生和重现传染病、肿瘤、心血管疾病、老年性疾病、精神性疾病及遗传病等理想的疾病动物模型，通过它们对分析人类疾病的致病机制和病因学，动物和人类行为，生物与环境相互作用，解答特定人群对某种疾病的易感性，以及研发新型特效预防和治疗药物均有重要推动作用^[55, 56]^。

## 6 展望

慢病毒载体在基因转染方面的独特优势，已经被广泛认可。今后的研究重点，将在以下几个方面：①进一步对慢病毒载体系统进行改进，或开发性的慢病毒体系，以使之适应不同的转基因需求；②如何进一步提高病毒的滴度，以消除技术上的瓶颈；③探索将慢病毒载体直接为临床所用时的安全性因素，以使其为患者所服务；④继续扩展慢病毒载体的使用范围。总之，作为一种有效的基因转染工具，慢病毒载体具有多方面的应用前景。
